# Dataset of the efficiency of the ultraviolet light activation of persulfate ion for the degradation of cobalt cyanocomplexes in synthetic mining wastewater

**DOI:** 10.1016/j.dib.2020.105346

**Published:** 2020-02-28

**Authors:** Samir Fernando Castilla-Acevedo, Luis Andrés Betancourt-Buitrago, Dionysios Demetriou Dionysiou, Fiderman Machuca-Martínez

**Affiliations:** a Natural and Exact Sciences Department, Universidad de la Costa, Calle 58 #55 – 66, 080002, Barranquilla, Colombia; b Escuela de Ingeniería Química, Universidad del Valle, Cali, Colombia; c Environmental Engineering and Science Program, Department of Chemical and Environmental Engineering, University of Cincinnati, Cincinnati, OH, 45221-0012, United States

**Keywords:** Advanced oxidation processes, UVC, Persulfate, Mining wastewater, Strong cyanocomplexes

## Abstract

In recent years, the extraction of gold has become important for the development of nations. However, mining wastewater represents an environmental problem due to its high content of free cyanide-based compounds and weak and strong cyanocomplexes for the use of sodium cyanide to obtain gold from minerals. The experimental data presented show the performance of the elimination of one of the strongest cyanocomplex that can appear in mining wastewater $\left({\left[\text{Co}{\left(\text{CN}\right)}_{6}\right]}^{3-}\right)$ by the ultraviolet C activation of persulfate (PS). The removal of total cobalt in solution was used as an indicator of the elimination of the cobalt cyanocomplexes that appear as transformation products from the degradation of ${\left[\text{Co}{\left(\text{CN}\right)}_{6}\right]}^{3-}$. The data evidence that strong cyanocomplexes can be eliminated from mining wastewater. The experimental runs were divided into two parts: as a first step, the influence of the UVC light was elucidated. Afterward, five initial concentrations of persulfate ion (0.1, 0.3, 0.5, 0.7 and 0.9 g/L of PS), two pH values (11 and 13) and two additional initial concentrations of contaminant (25 mg/L and 75 mg/L of ${\left[\text{Co}{\left(\text{CN}\right)}_{6}\right]}^{3-}$ ) were examined to find the optimal parameter where the highest Co removal is obtained.

**Table:** Specifications Table

Subject	Environmental science
Specific subject area	Industrial and domestic wastewater treatment by advanced oxidation processes (AOPs).
Type of data	Figures
How data were acquired	Data were obtained by the measurement of total cobalt dissolved in solution using a Thermo iCE 3000 Series atomic absorption spectrometer. A Jasco V730 spectrometer was used for the spectra measurement and to measure the concentration of ${\text{CNO}}^{-}$ and ${\text{NO}}_{3}^{-}$. The ${\text{CN}}^{-}$ concentration was followed using volumetric titration as it is specified in the standard methods 4500-CN.
Data format	Raw Analyzed
Parameters for data collection	The initial concentration of persulfate ion, the pH value and the initial concentration of the contaminant were the experimental parameters to evaluate the performance of the ultraviolet activation of PS for the elimination of ${\left[\text{Co}{\left(\text{CN}\right)}_{6}\right]}^{3-}$.
Description of data collection	All the experimental data were collected at lab-scale
Data source location	Colombia
Data accessibility	Within the article

**Table:** 

**Value of the Data** • The data offer a systematic way of optimizing the performance of persulfate according to the chemical properties and its interactions with the UV light. Besides, it presents the fundamental criteria to operate the process at maximum efficiency, allowing comparisons of operational conditions during the cobalt cyanocomplexes treatment. • The dataset is useful and novel since, before this work, the degradation of any cyanocomplex had not been reported in the literature using UVC/PS. • As cobalt cyanocomplexes degradation by the activation of persulfate has not been reported in the literature. This dataset represents a benchmark for the development of further experiments to optimize AOPs for the elimination of mining-related compounds. • This data can be used to develop kinetic and optimization models for the improvement of the technology used in the elimination of cyanide-based compounds in mining wastewater. • The data is useful for scaling up and economic analysis for mining wastewater treatment.

## 1 Data description

**Fig. 1:**
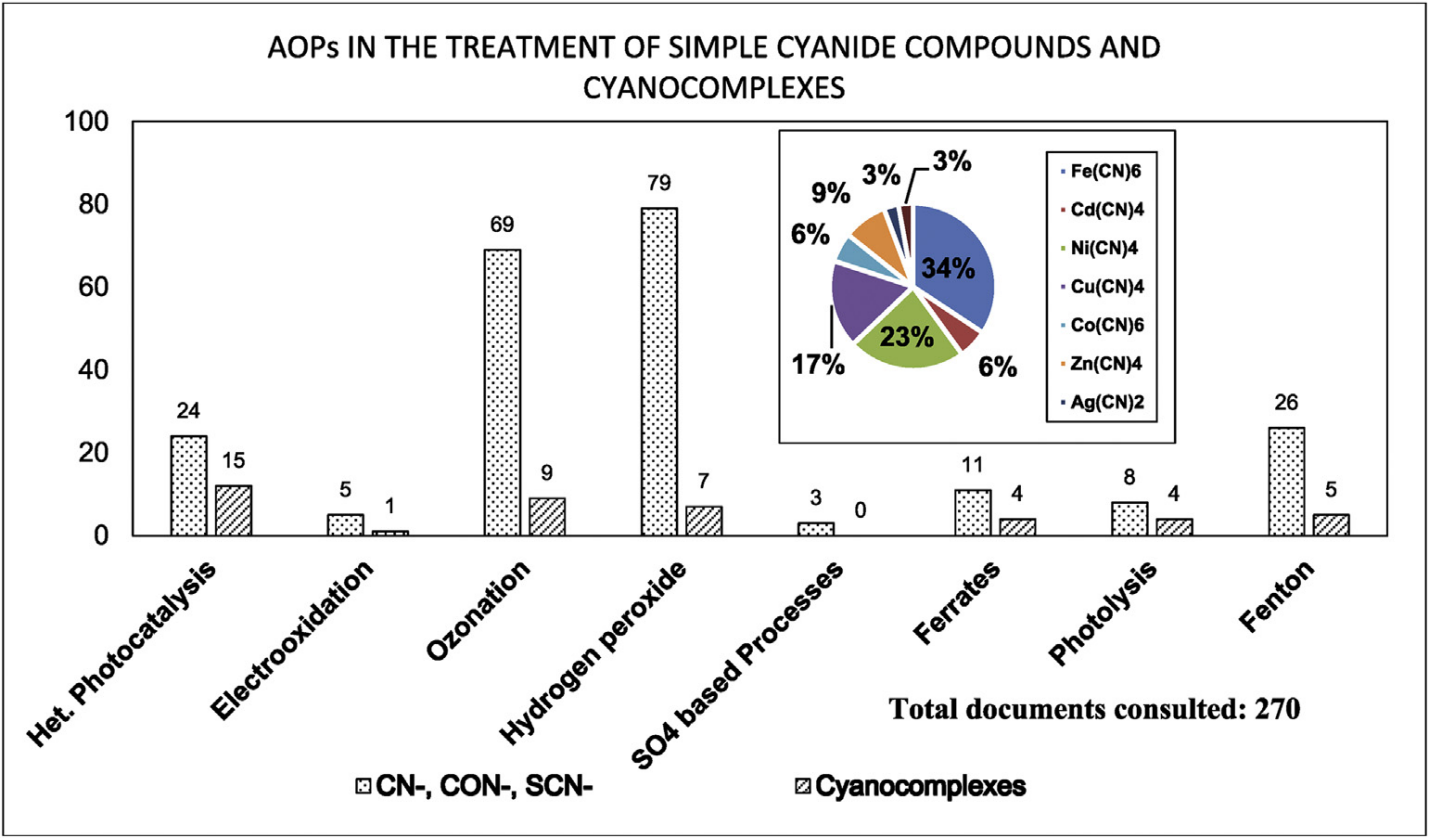
Results of the search in the SCOPUS database. Search equations: “AOPs” and “cyanide complexes” “water treatment” and “AOPs” and “cyanide” and “water treatment”, where AOPs, corresponds to the technology reviewed. For example, ozone, ozonation, Fenton, hydrogen peroxide, etc. Search date: November 5th, 2019.

**Fig. 2:**
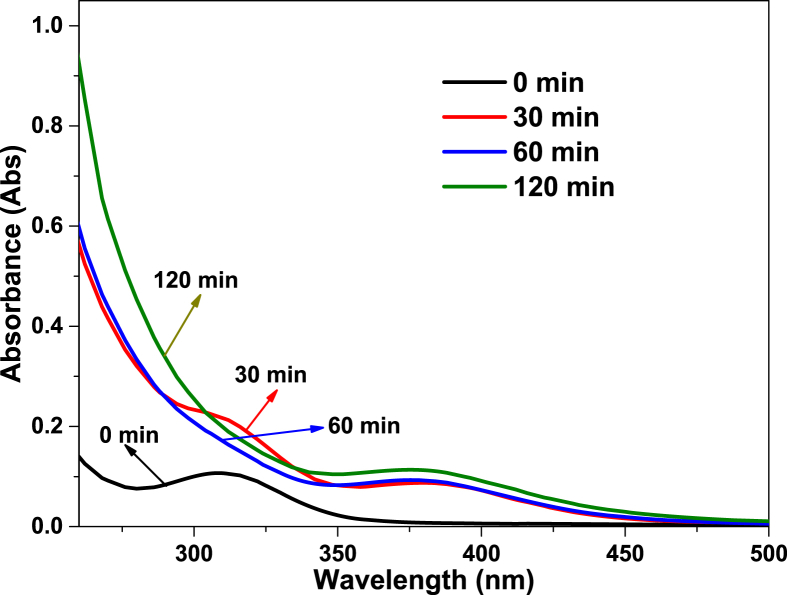
Spectra measurement during the photolysis process over time.

**Fig. 3:**
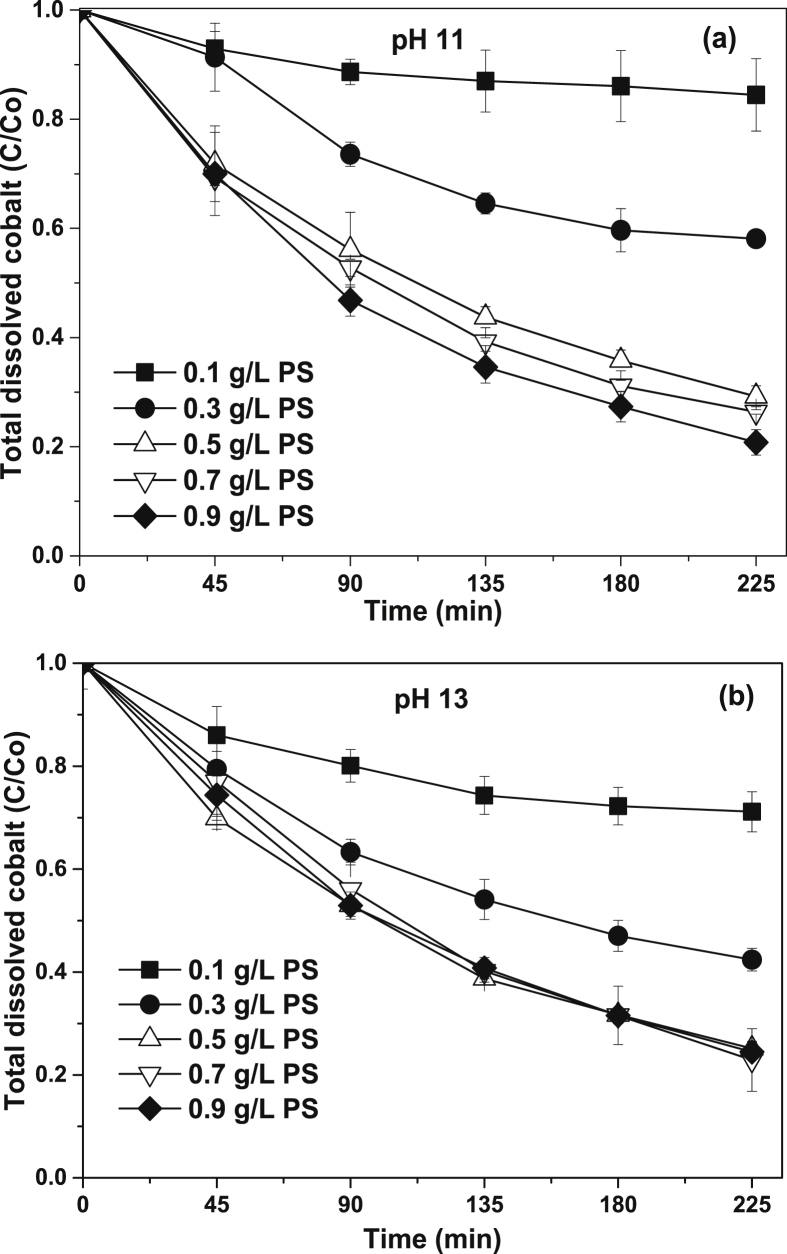
Performance of the elimination of Co by $\text{U}\text{V}\text{C}/{\text{S}}_{2}{\text{O}}_{8}^{2-}$ a) at pH 11; b) at pH 13.

**Fig. 4:**
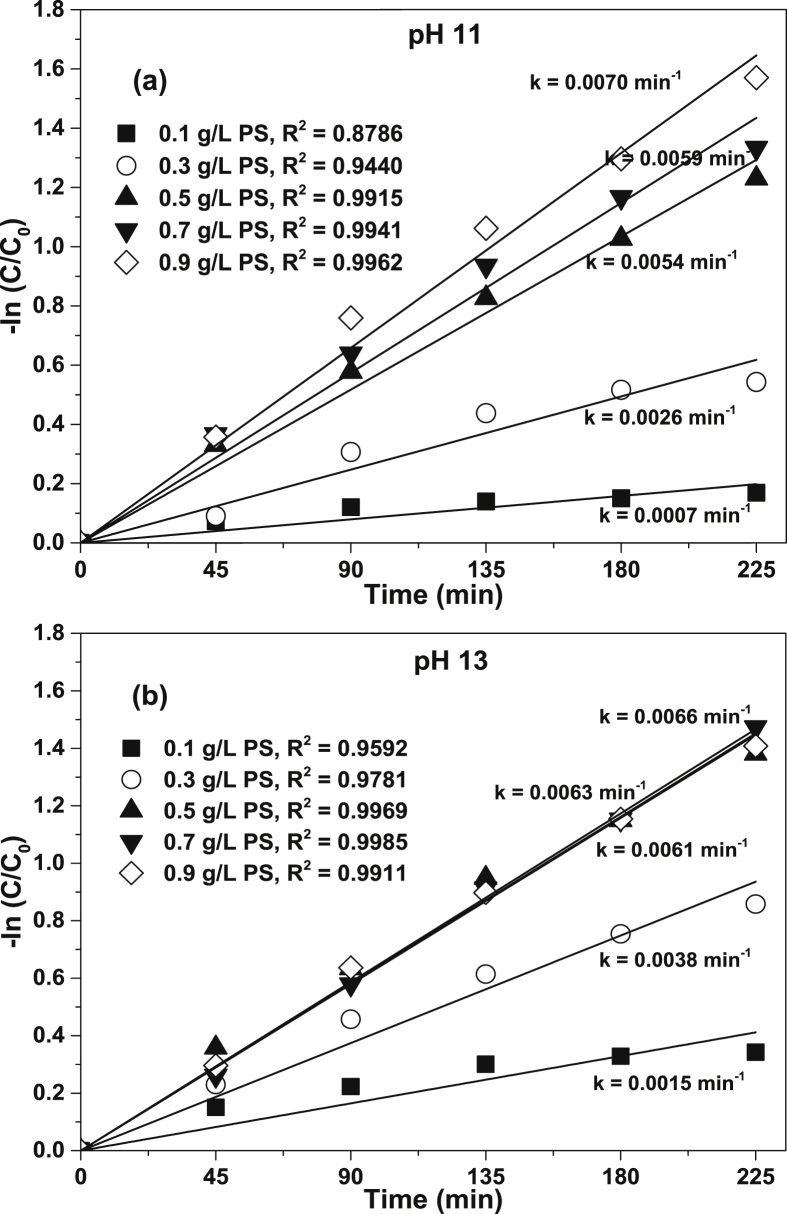
Data and fitting curves of pseudo first order kinetics: a) pH 11; b) pH 13.

**Fig. 5:**
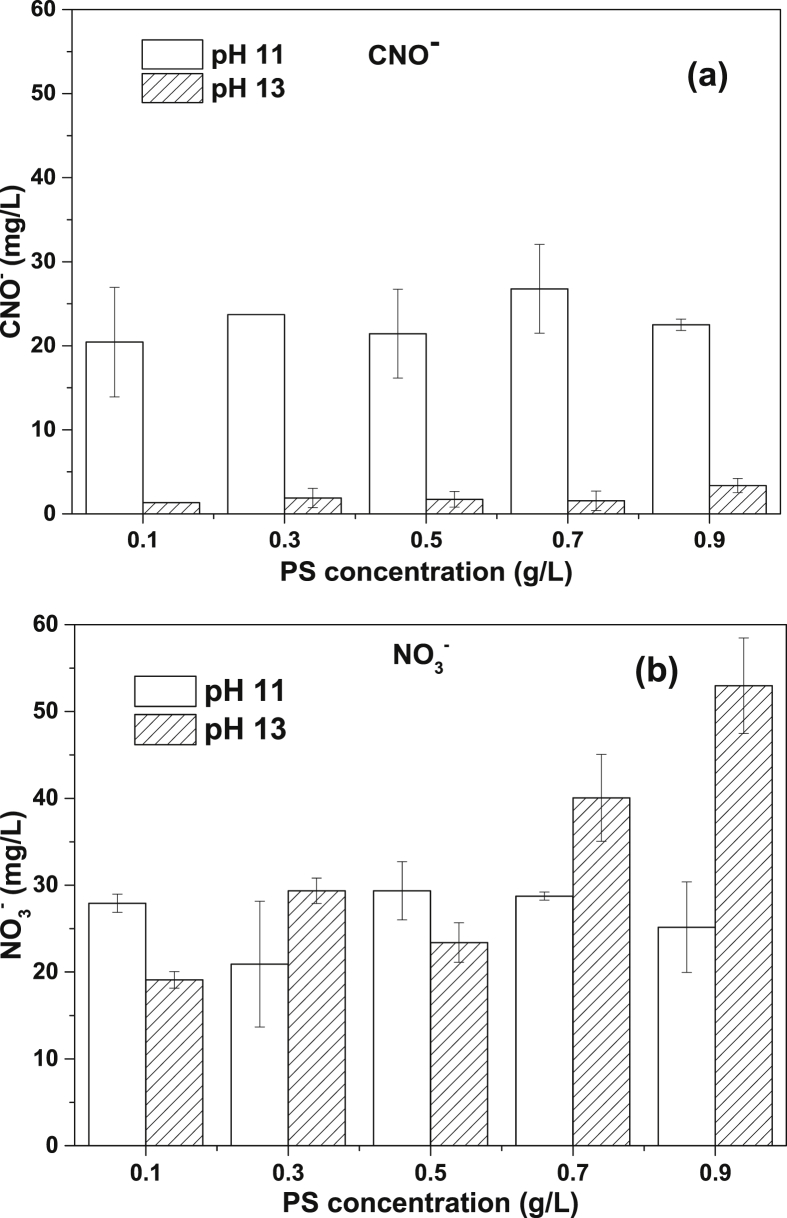
Concentration of some transformation products in all the tests made at oxic conditions. a)${\mathrm{CNO}}^{-}$; b) ${\mathrm{NO}}_{3}^{-}$.

**Fig. 6:**
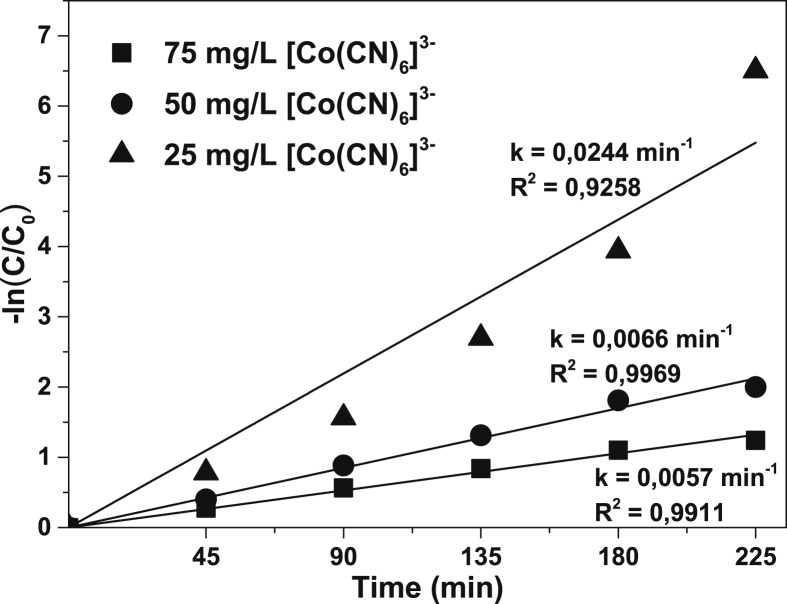
Pseudo first order-rate constants and fitting of apparent kinetics at different initial concentrations of ${\left[\text{C}\text{o}{\left(\text{C}\text{N}\right)}_{6}\right]}^{3-}$. Operating conditions: 0.5 g/L of PS and 50 mg/L of ${\left[\mathrm{Co}{\left(\mathrm{CN}\right)}_{6}\right]}^{3-}$.

Fig. 1 shows the literature search made to show the state of the art of the advanced oxidation processes applied for the degradation of simple ${\text{CN}}^{-}$-based compounds, weak and strong cyanocomplexes. Based on our knowledge there is not any scientific report about the degradation of cyanocomplexes using the ultraviolet (UVC) activation of persulfate. During the photolysis process, the spectra measurement of the contaminant solution was carried out to elucidate the possible ${\left[\text{Co}{\left(\text{CN}\right)}_{6}\right]}^{3-}$ degradation mechanism, which is described in Fig. 2. After the photolysis test, experimental runs with the five initial concentrations of ${\text{S}}_{2}{\text{O}}_{8}^{2-}$ (0.1, 0.3, 0.5, 0.7 and 0.9 g/L of PS) and the two pH values (11 and 13) were made to find the optimal operating conditions to eliminate the cobalt cyanocomplex. The data of the UVC/PS efficiency can be observed in Fig. 3a and b. Later, the performance of the UVC activation of PS was adjusted to a Pseudo-first-order kinetic to elucidate the kinetic constants. The data obtained were plotted in Fig. 4a and b. Simultaneously, ${\text{CNO}}^{-}$, and nitrate concentrations were measured to verify if the toxicity of the water treated decreases after the treatment, those experimental data are shown in Fig. 5a and b. Finally, tests at a fixed concentration of PS (0.5 g/L) and pH 13 were made to evaluate the influence of the initial contaminant concentration. The data obtained were adjusted to a Pseudo-first-order kinetic and it is observed in Fig. 6 along with the kinetic constants calculated.

## 2 Experimental design, materials, and methods

All the reagents and materials used in the present work are shown in Table 1. The reagents were used as received without further purification.

**Table 1:** Reagents and materials used for the experimental tests and analysis.

Material (purity)	Brand	Application
Potassium hexacyanocobaltate (97%)	Sigma Aldrich	Contaminant precursor
Sodium Hydroxide (97%)	Carlo Erba	pH adjustment
Sodium persulfate (97%)	AppliChem Panreac	Oxidant agent
Sulfuric Acid (96%)	Fisher Scientific	pH adjustment
Silver nitrate (99%)	Merck	Titrating agent
p-dimethylaminobenzalrodanine (99%)	Fisher Scientific	Indicator
Aminobenzoic acid (99%)	Fisher Scientific	Measurement of ${\text{CNO}}^{-}$ concentration
Hydrochloric acid (37%)	Fisher Scientific	Measurement of ${\text{CNO}}^{-}$ and $NO_{3}^{-}$ concentration
Borosilicate glass bubbler	Pyrex	Experimental set up
Multiparameter	Orion 4 star (ORION 083005MD)	pH measurements
Steel cylindrical reactor vessel	–	Experimental set up
Centrifugal Pump	–	Experimental set up
Centrifuge	Champion (S-33)	Experimental tests
UV–Vis spectrophotometer	Jasco (V 730)	Measurements
Atomic absorption spectrometer	Thermo iCE 3000 Series	Measurements
Analytical balance	Radwag (AS 310R2)	Measurement

**Fig. 7:**
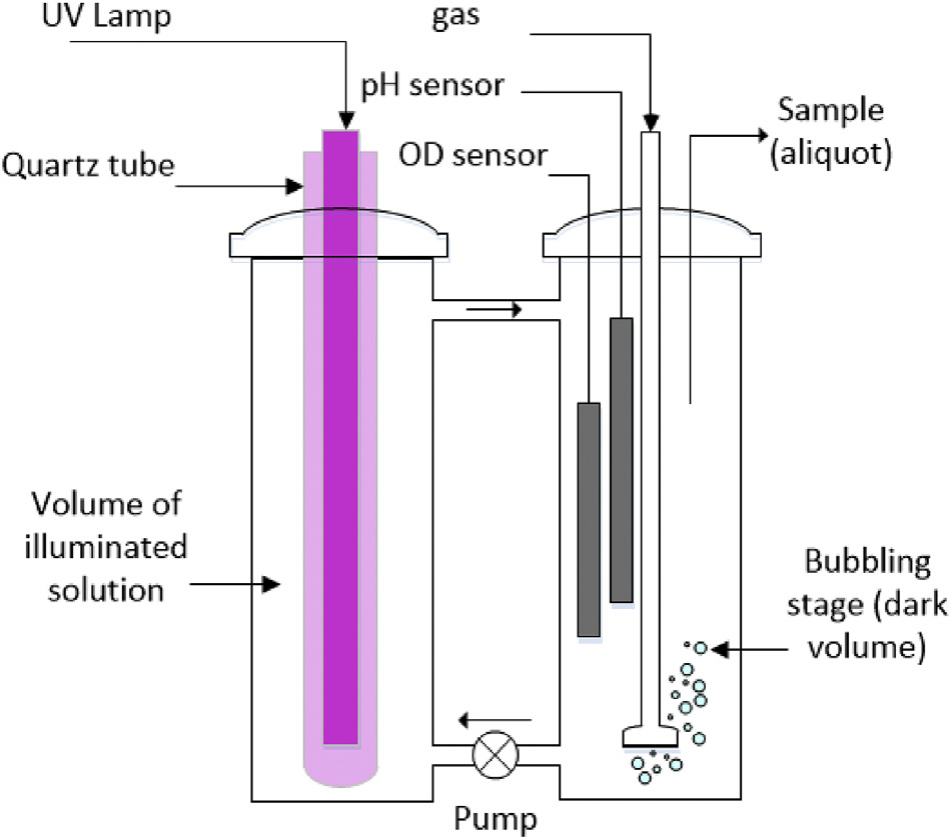
Experimental set up for the degradation tests.

Since the solution pH must be above 10 to avoid the volatilization of free cyanide [[1], [2], [3]], deionized water type II and NaOH 10 M were used to prepare in advance the stock solutions of 2 g/L of ${\text{S}}_{2}{\text{O}}_{8}^{2-}$ and 1 g/L ${\left[\text{Co}{\left(\text{CN}\right)}_{6}\right]}^{3-}$. Then, specific aliquots were taken to prepare the solutions at the initial concentrations required. NaOH 10 M and ${\text{H}}_{2}{\text{SO}}_{4}$ 5 M were used to control the solution pH during the treatment. An ultraviolet-C (253.7 nm) 6W low-pressure Hg lamp was used to irradiate the steel cylindrical reactor vessel which is connected to a bubbler vessel and to a centrifugal pump as it is observed in Fig. 7. Since the solution is recirculated along the reaction time, the experimental system was operated under constant stirring conditions. The lamp was connected 30 minutes before to start the experimental runs to stabilize the emission of photons. Later, the light source was placed into a cylindrical quartz vessel (quartz tube, 23 cm in height and 2.31 cm in external diameter) and put into the reactor to initiate the degradation tests. The reaction volume was 800 mL and, each experiment was carried out at least by duplicate and the average value was reported along with its standard deviation. The removal of total cobalt in solution was used as an indicator of the elimination of the cobalt cyanocomplexes that may appear as transformation products from the degradation of ${\left[\text{Co}{\left(\text{CN}\right)}_{6}\right]}^{3-}$.

As a first step, the photolysis process was carried out. 50 mg/L of ${\left[\text{Co}{\left(\text{CN}\right)}_{6}\right]}^{3-}$ were added to the reaction system in the absence of ${\text{S}}_{2}{\text{O}}_{8}^{2-}$ and, specific aliquots of 10 mL were taken with a syringe every 30 minutes during 2 hours of treatment. Changes in the pH value were not allowed by more than ±0.3 units. With the samples obtained over time, total dissolved cobalt, ${\text{CN}}^{-}$, ${\text{CNO}}^{-}$, and ${\text{NO}}_{3}^{-}$ were measured to evaluate the performance of the photolysis test.

Two pH values (11 and 13) and five initial concentrations of PS (0.1, 0.3, 0.5, 0.7 and 0.9 g/L) were used for the second set of experiments to evaluate the efficiency of UVC/PS in the degradation of 50 mg/L of ${\left[\text{Co}{\left(\text{CN}\right)}_{6}\right]}^{3-}$. After obtaining the optimal value of pH and initial concentration of oxidant where the highest Co removal was obtained, additional experiments to evaluate the influence of the initial concentration of the contaminant were performed using 25 mg/L and 75 mg/L of ${\left[\text{Co}{\left(\text{CN}\right)}_{6}\right]}^{3-}$. The dissolved oxygen was measured during the reaction using an ORION 4 start (083005MD) multiparameter to ensure reproducible data. Samples were taken every 45 minutes during 225 minutes of reaction and those were centrifuged and filtered to remove the cobalt precipitated that appeared during the degradation process.

Afterward, total dissolved cobalt was measured using a Thermo iCE 3000 series atomic absorption spectrometer. Spectra measurements using a Jasco V730 UV–Vis spectrometer were made as well to determine possible changes and transformations of the contaminant of interest. Since the degradation of the cobalt cyanocomplex leads to the release of free cyanide which in turn is oxidized to ${\text{CNO}}^{-}$ and ${\text{NO}}_{3}^{-}$ [[5], [6], [7]], those compounds were measured as another indicator of the degradation of ${\left[\text{Co}{\left(\text{CN}\right)}_{6}\right]}^{3-}$. Thus, Free cyanide concentration was determined by titration using the methodology reported by the standard methods 4500-CN [8]. 4 mL of the sample was diluted in 10 mL of deionized water type II. Two drops of p-dimethylaminobenzalrodanine (indicator) and the amount required of 1.8 mM of ${\text{AgNO}}_{3}$ were added until obtain the desired change of color. The ${\text{CNO}}^{-}$ concentration was tracked using 2-aminobenzoic acid 0.01 M [9]. 1 mL of the previous compound was added to 1 mL of sample and it was heated at 40 °C for 10 min to add subsequently 2 mL of HCl 6 N. The solution was mixed and heated again at 90 °C for 15 minutes and finally it was measured at 310 nm in the UV–Vis spectrometer. The ${\text{CNO}}^{-}$ concentration was calculated according to stoichiometry. Finally, ${\text{NO}}_{3}^{-}$ concentrations was measured adding 40 μL of HCl 1 N to 2 mL of sample and measuring the resultant solution at 220 nm in the UV–Vis spectrometer [10].

## References

[1] Kuyucak N., Akcil A. (2013). Cyanide and removal options from effluents in gold mining and metallurgical processes. Miner. Eng..

[2] Dai X., Simons A., Breuer P. (2012). A review of copper cyanide recovery technologies for the cyanidation of copper containing gold ores. Miner. Eng..

[3] Johnson C.A., Grimes D.J., Leinz R.W., Rye R.O. (2008). Cyanide speciation at four gold leach operations undergoing remediation. Environ. Sci. Technol..

[5] Kim S.H., Lee S.W., Lee G.M., Lee B.T., Yun S.T., Kim S.O. (2016). Monitoring of TiO2-catalytic UV-LED photo-oxidation of cyanide contained in mine wastewater and leachate. Chemosphere.

[6] Aguado J., Van Grieken R., López-Muñoz M.J., Marugán J. (2002). Removal of cyanides in wastewater by supported TiO2-based photocatalysts. Catal. Today.

[7] Augugliaro V., Loddo V., Marcì G., Palmisano L., López-Muñoz M.J. (1997). Photocatalytic oxidation of cyanides in aqueous titanium dioxide suspensions. J. Catal..

[8] W.E.F. American Public Health Association, American Water Works Association (1998). Standard Methods for the Examination of Water and Wastewater.

[9] Guilloton M., Karst F. (1985). A spectrophotometric determination of cyanate using reaction with 2-aminobenzoic acid. Anal. Biochem..

[10] APHA, AWWA, WEF (1998). Standard Methods for the Examination of Water and Wastewater, Washington DC, USA.

